# Association of Elevated Galectin-4 Concentrations with Obesity, Diabetes, and Cardiovascular Diseases

**DOI:** 10.3390/ijms26199402

**Published:** 2025-09-26

**Authors:** Krystian Kozak, Monika Zajkowska

**Affiliations:** 1 ALAB Laboratoria sp. z o.o., Stępińska 22/30, 00-739 Warsaw, Poland; krysmir12@gmail.com; 2 Department of Neurodegeneration Diagnostics, Medical University of Białystok, 15-269 Białystok, Poland; 3 Department of Biochemical Diagnostics, Medical University of Bialystok Clinical Hospital, 15-269 Białystok, Poland

**Keywords:** Gal-4, CVD, biomarker, inflammation, gut microbiota

## Abstract

Obesity, type 2 diabetes mellitus (T2DM), and cardiovascular diseases (CVDs) represent major global health burdens with overlapping pathophysiological mechanisms, including chronic low-grade inflammation, oxidative stress, and gut microbiota dysbiosis. Galectins, a family of β-galactoside-binding lectins, have been implicated in immune regulation, inflammation, and tissue remodeling. Among them, Galectin-4 (Gal-4), primarily expressed in the gastrointestinal tract, has emerged as a potential biomarker due to its roles in epithelial integrity, inflammatory signaling, and metabolic regulation. Despite its established involvement in cancer and inflammatory disease, the relevance of Gal-4 in cardiometabolic disorders remains poorly defined. A comprehensive literature search was conducted via the PubMed and ScienceDirect databases. The association between Gal-4 and obesity has been reported, indicating that elevated Gal-4 levels correlate with obesity, but primarily in individuals with diabetes. Circulating Gal-4 concentrations are consistently elevated in diabetic populations. In CVD, elevated Gal-4 levels are associated with ischemic heart disease, heart failure, aortic stenosis, carotid atherosclerosis, and adverse outcomes following myocardial infarction and stroke. Furthermore, prospective studies link Gal-4 to increased risk of cardiovascular events and mortality, underscoring its potential prognostic relevance. Available evidence regarding the mechanistic role of Gal-4 in the pathogenesis of obesity, diabetes, and cardiovascular disease remains limited; therefore, future studies should address whether Gal-4 actively contributes to cardiometabolic dysfunction or only reflects secondary inflammatory or fibrotic processes. Elucidating the biological functions of Gal-4 may provide insight into its utility in diagnostics and support the development of novel therapeutic strategies for cardiometabolic disorders.

## 1. Introduction

### 1.1. Obesity

**Figure 1:**
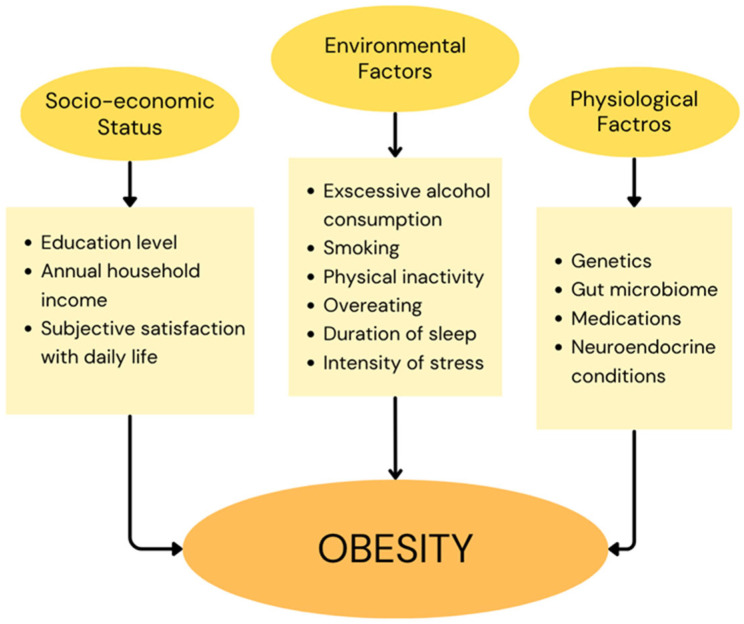
Risk factors associated with the development of obesity.

Obesity is consistently considered one of the greatest threats to global health, leading to various complications such as cardiovascular diseases, type 2 diabetes, asthma, and even cancers [1]. According to WHO ‘obesity is a chronic complex disease defined by excessive fat deposits that can impair health’. The most commonly used measure of obesity is the body mass index (BMI) comprising a person’s weight and height expressed in kilograms per meter squared [2]. It is estimated that over 1 billion people will suffer from obesity by 2030 [3]. Obesity-related complications can lead to a significant reduction in life expectancy, with some studies reporting up to 30 years of life lost by individuals with a BMI over 45 kg/m^2^, depending on demographic and clinical factors [4]. While obesity is most often attributed to an imbalance between energy intake and energy expenditure, its underlying etiology is highly complex. It involves a multifaceted interplay of genetic, physiological, environmental, psychological, social, economic determinants that collectively contribute to its development and persistence [5]. Specific lifestyle behaviors such as excessive alcohol consumption, smoking, physical inactivity, and overeating have been identified as direct contributors to the development of obesity [1]. Additional factors associated with obesity include education level, duration of sleep, intensity of stress, annual household income, subjective satisfaction with daily life, gut microbiome, use of medications, and neuroendocrine conditions [6,7]. Key risk factors for obesity are summarized in Figure 1.

In roughly 5% of cases, obesity can be directly linked to specific genetic mutations, a form classified as Mendelian or monogenic obesity. Most commonly, these mutations affect hormonal pathways such as the leptin–melanocortin signaling axis in the hypothalamus, which regulates appetite and energy balance. Additionally, recent findings indicate that pathways involved in adipocyte differentiation may also be disrupted in this form of obesity [8]. Inflammation appears to play a critical role in the development and progression of obesity. Obesity-induced inflammation is fundamentally driven by metabolic excess, with the overconsumption of nutrients serving as the initial trigger. Adipocytes are both the targets of nutrient-induced stress and the initiators of downstream inflammatory cascades, ultimately disrupting metabolic homeostasis [9]. Within adipose tissue, inflammatory mediators contribute to the development of insulin resistance by impairing insulin signaling in adipocytes and reducing glucose uptake [10]. This decline in insulin sensitivity extends to the liver, where one of the key consequences is the dysregulation of gluconeogenesis suppression, contributing to hyperglycemia [11]. Comparative studies have shown significant differences in the gut microbiota composition between lean and obese hosts. Obesity was associated with a decrease in *Bacteroidetes* and increase in *Firmicutes*. Notably, weight loss can reverse this microbial imbalance. The altered gut microbiota in obesity may also be implicated in weight gain. Transplantation of microbiota from obese to lean mice induced greater weight gain [12,13]. Beyond microbial and hormonal contributors, organelle dysfunction within metabolic cells plays a critical role in propagating both inflammation and metabolic deterioration. Excess nutrient supply imposes considerable stress on cellular organelles, particularly endoplasmic reticulum. This organelle stress serves as a proximal signal that initiates inflammatory responses, even before specific cytokines or lipotoxic mediators are fully engaged [14]. Besides insulin resistance and hyperinsulinemia, obesity is linked to numerous diseases and metabolic disturbances associated with high morbidity and mortality, including hypertension, gallbladder disease, cancer, coronary heart disease, elevated all-cause mortality, and type 2 diabetes [15]. The molecular mechanisms by which obesity exerts its impairments on overall patient health, considering the heterogeneity of obesity, remain to be fully understood [16]. Given the wide range of physiological disruptions associated with obesity, one of the most prominent and closely linked conditions is type 2 diabetes.

### 1.2. Diabetes

**Figure 2:**
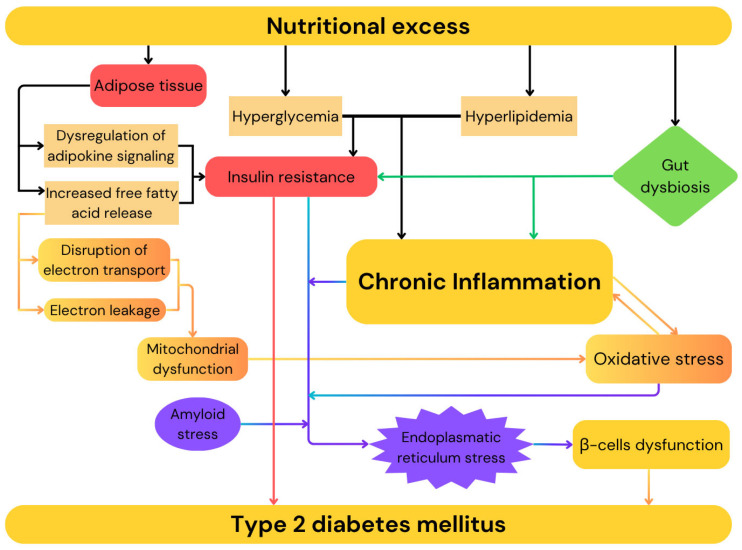
Mechanisms contributing to the development of type 2 diabetes mellitus.

Type 2 diabetes mellitus (T2DM) is characterized by chronic hyperglycemia resulting from a combination of insulin resistance and impaired insulin secretion. The prevalence of T2DM continues to rise globally, mirroring the increasing rates of obesity. The global prevalence of diabetes has quadrupled over the past three decades and is estimated to reach 700 million individuals by 2045. Type 2 diabetes constitutes over 90% of diabetes mellitus cases [17]. The global surge in obesity, physical inactivity, high-calorie diets, and an aging population are the primary factors driving the type 2 diabetes epidemic [18]. This disease is a heterogeneous metabolic disorder, defined by insufficient insulin secretion from pancreatic islet β-cells in the presence of peripheral insulin resistance [19]. As the disease progresses, insulin secretion becomes insufficient to sustain glucose homeostasis, resulting in chronic hyperglycemia. Individuals affected by type 2 diabetes are typically obese or have elevated body fat percentage, localized mainly in the abdominal region. Under these conditions, adipose tissue contributes to the development of insulin resistance through multiple pro-inflammatory pathways, including increased free fatty acid release and dysregulation of adipokine signaling [20]. While β-cell dysfunction in type 2 diabetes has traditionally been attributed to β-cell death, emerging evidence indicates that it results form a complex interplay between environmental factors and multiple molecular pathways involved in cellular homeostasis. In context of nutritional excess, as seen in obesity, the presence of hyperglycemia and hyperlipidemia promotes insulin resistance and chronic inflammation, exposing genetically susceptible β-cells to a range of toxic pressures including inflammation, endoplasmic reticulum stress, and metabolic, oxidative, and amyloid stress, which may ultimately compromise islet integrity [21,22]. Elevated free fatty acids and glucose concentration promote β-cells dysfunction by triggering endoplasmic reticulum stress and activating pro-apoptotic unfolded protein response pathways, primarily as a result of lipotoxic and glucotoxic effects. This dysfunction is mediated by disturbances in calcium signaling, the buildup of misfolded proteins such as proinsulin and islet amyloid polypeptide, enhanced production of reactive oxygen species, and interleukin-1β-driven macrophage recruitment [22,23]. Moreover, elevated circulating free fatty acids contribute to mitochondrial dysfunction via two primary mechanisms: metabolic by-products of FFA oxidation disrupt electron transport within the mitochondrial respiratory chain and the integration of FFAs into mitochondrial membranes likely promotes electron leakage [24]. Emerging evidence indicates that microbial dysbiosis may contribute to insulin resistance and the onset of type 2 diabetes. High-fat diets have been shown to markedly increase lipopolysaccharide production form Gram-negative bacteria, promoting chronic low-grade inflammation and insulin resistance. Additionally, dysbiosis can impair the synthesis of short-chain fatty acids, which are essential for maintaining gut barrier integrity, supporting pancreatic β-cells proliferation, and enhancing insulin production. Altered microbial composition may also disrupt the generation of other key metabolites, including branched-chain amino acids and trimethylamine, thereby impairing glucose homeostasis and facilitating diabetes development [25,26,27,28,29,30]. The key mechanisms contributing to the development of T2DM are summarized in Figure 2.

Type 2 diabetes leads to a wide range of chronic and acute complications including hypertension, non-alcoholic fatty liver disease, neuropathy, retinopathy, nephropathy, diabetic foot disorder, various cancers, peripheral artery disease, angina pectoris, heart failure, myocardial infarction, and stroke [31,32,33,34]. Considering the high morbidity and mortality associated with complications of poorly controlled type 2 diabetes, early diagnosis is critical to enable timely intervention and the risk of disease progression [35]. Criteria for the diagnosis of diabetes and prediabetes are shown in Table 1.

Cardiovascular disease represents the leading cause of morbidity and mortality in individuals with type 2 diabetes and necessitates rigorous control of glycemia, lipid levels, and blood pressure to reduce the risk of complications and slow disease progression. Intensive glycemic management show significant benefits in mitigating microvascular complplications, including retinopathy, neuropathy, and nephropathy [18]. In light of the significant overlap between type 2 diabetes and cardiovascular disease, understanding their interconnected pathophysiology is essential for improving clinical outcomes.

### 1.3. Cardiovascular Diseases

Cardiovascular diseases (CVDs) encompass a broad range of cardiac and vascular conditions, including heart failure, stroke, ischemic heart disease and peripheral arterial disease, all of which occur at substantially higher rates in individuals with type 2 diabetes [36]. Over the past three decades, the number of prevalent cases of CVDs has nearly doubled, reaching 523 million in 2019 and constituting the leading cause of global disease burden, resulting in an estimated 18.6 million deaths [37]. Ischemic heart disease is a major cause of acute myocardial infarction, which has an out-of-hospital survival rate of only 10.6% [38]. Given the high morbidity and mortality associated with CVDs, recent years have seen increasing efforts to identify novel biomarkers for more accurate diagnosis [39]. A multi-biomarker approach incorporating markers such as high sensitivity C-reactive protein (hs-CRP), cardiac troponin I (cTnI), *N*-terminal pro-B-type natriuretic peptide (NT-proBNP), heart-type fatty acid-binding protein (H-FABP), fibroblast growth factors 19 and 21 (FGF19, FGF21), retinol-binding protein 4 (RBP4), among others may prove useful in improving diagnostic accuracy [40].

The development of cardiovascular diseases is driven by a complex interplay of molecular, cellular, and systemic mechanisms. The most significant and widely studied pathophysiological mechanisms include hypertension, obesity, diabetes, chronic inflammation, oxidative stress, calcium mishandling, platelet dysfunction, and insulin growth factor 1 pathway [41,42,43,44]. Endothelial dysfunction plays a central role in the development of atherosclerosis by reducing nitric oxide (NO) bioavailability in response to cardiovascular risk factors, infections, and environmental stress, thereby impairing vascular homeostasis and increasing endothelial permeability. These alternations allow cholesterol-containing lipoproteins, particularly low-density lipoprotein cholesterol (LDL-C), to accumulate in the subendothelial space, where they undergo oxidative modification and aggregation [45,46,47]. Inflammatory conditions further promote the formation of small dense LDL-C particles, which have prolonged circulation time, increased wall penetration, and heightened susceptibility to oxidation [47]. The oxidized LDL-C acts as a potent pro-inflammatory stimulus, activating endothelial cells and initiating a cascade of immune responses that contribute to the development and progression of atherosclerosis. In response to modified lipoproteins in subendothelial space, circulating monocytes adhere to activated endothelial cells, migrate into the intima, and differentiate into macrophages [48]. Depending on the local microenvironment macrophages differentiate into either pro-inflammatory M1 or anti-inflammatory M2, with T lymphocytes promoting M1 polarization that contributes to atherosclerotic progression. Concurrently, activated endothelial cells and macrophages upregulate the expression of adhesion molecules such as VCAM-1, ICAM-1, and E-selectin, along with pro-inflammatory cytokines, thereby amplifying leukocyte recruitment and sustaining vascular inflammation [48,49]. The atherosclerotic plaque is composed of lipid deposits, various cell types including vascular smooth muscle cells (VSMCs), inflammatory cells, macrophages, and endothelial cells, as well as extracellular matrix, and chemokines. A necrotic core is typically covered by a fibrous cap rich in type I collagen, synthesized predominantly by VSMCs that have migrated into the intima in response to cytokines and matrix metallopeptidases [49].

Recent studies demonstrate a strong link between gut microbiota-derived metabolites, trimethylamine *N*-oxide (TMAO) and phenylacetylglutamine (PAG), and cardiovascular disease. TMAO contributes to atherosclerosis by impairing reverse cholesterol transport, altering sterol metabolism, and disrupting bile acid homeostasis. PAG, produced during phenylalanine metabolism by gut microbes, interacts with G-protein-coupled receptors, particularly α- and β-adrenergic receptors (ARs), which play key roles in platelet function and cardiovascular regulation. Gut microbiota can modulate AR activity, thereby influencing cardiovascular phenotypes. The absence of gut microbiota disrupts cardiovascular homeostasis, thrombosis, and blood pressure regulation [50].

Although obesity, diabetes, and cardiovascular diseases arise from diverse etiologies, chronic low-grade inflammation and oxidative stress appear to constitute common underlying mechanisms contributing to the pathogenesis of all three conditions. Moreover, disturbances in gut microbiota composition have also been increasingly recognized as a shared feature that may exacerbate metabolic and cardiovascular dysfunction in these disorders. In recent years, growing attention has been directed toward endogenous lectins, particularly Galectins, as potential modulators of inflammatory and immune responses in cardiometabolic disease.

### 1.4. Galectins

Galectins are a family of β-galactoside-binding proteins involved in cell adhesion, apoptosis, immune regulation, and fibrosis which belongs to the group called Lectins. Lectins are a diverse group of proteins, other than immunoglobulins, that can selectively and reversibly bind to specific carbohydrate structures, without altering the chemical composition of the sugars [51]. Lectins can be classified by their subcellular localization or by their structural characteristics. Structural classification includes C-type lectins (calcium-dependent), I-type lectins (with immunoglobulin-like domains), P-type lectins (mannose-6-phosphate specific), pentraxins (pentameric), and S-type lectins [52]. Genes encoding lectins have been identified across all domains of life; however, the first lectin to be discovered was isolated from *Ricinus communis* in 1888 [53,54]. The widespread presence of lectins across diverse organisms and within various cellular compartments, both intra- and extracellularly, reflects their involvement in a broad spectrum of biological processes, including bacterial adhesion, cell proliferation and immune defense [55,56,57]. Although the interaction between a single carbohydrate recognition domain (CRD) of a lectin and its glycan ligand is often characterized by low affinity, the multivalency of most lectins (through the presence of multiple CRDs) enables cooperative binding, thereby markedly enhancing overall avidity toward glycosylated targets [54,58]. This multivalent binding capacity not only strengthens lectin-ligand interactions but also underlines the high degree of specificity that lectins exhibit toward distinct glycan epitopes, which are frequently expressed on the surfaces of various pathogens, including viruses, as well as malignant cells [54,58]. Owing to this specificity, lectins emerged as promising candidates for diagnostic and therapeutic applications, particularly in the context of pathogen neutralization and targeted drug delivery [54]. S-type lectins, known as Galectins, form a superfamily of proteins that bind β-galactosides. Galectins were initially referred to as S-type lectins to indicate their sulfhydryl dependency and the presence of free cysteine residues. However, many subsequently discovered galectins contain variable numbers of free cysteine residues or lack them entirely, and site-directed mutagenesis later revealed that cysteine residues are not involved in the saccharide binding [59,60]. Galectins represent the most broadly expressed class of lectins across all organisms and are structurally categorized into three main types: prototypical, chimeric, and tandem-repeat [52]. Galectins modulate cellular behavior through interactions influenced by cell-specific glycan signatures. Lactose and *N*-acetyllactosamine (LacNAc) are canonical ligands of Galectins, but these proteins can recognize multiple LacNAc units present in *N*- and *O*-glycans on cell surface glycoconjugates, including [-3Galβ1-4GlcNAcβ1-]*_n_* (poly-*N*-acetyllactosamine). Modifications of LacNAc or poly-LacNAc structures may alter galectin-glycan interactions, for example, addition of a terminal sialic acid or an internal fucose residue to the LacNAc sequence may result in loss of LacNAc binding by some Galectins [60,61,62]. Unlike most glycan-binding proteins, Galectins also function intracellularly, where they can engage both glycan-dependent and glycan-independent mechanisms, notably forming signaling platforms in response to endolysosomal damage. Although Galectins are broadly expressed and secreted through non-classical pathways, their activity is tightly regulated via spatial, temporal, and environmental mechanisms such as oxidative inactivation or proteolysis [63]. They play key roles in regulating immune responses and driving inflammatory processes. Moreover, they are critically implicated in tumor biology, contributing to cancer cell invasion, progression, metastasis, and the formation of new blood vessels (angiogenesis) [52]. Table 2 presents a comprehensive classification of Galectins.

Galectin-4 (Gal-4), primarily expressed in the gastrointestinal tract, has emerged as a candidate biomarker and effector molecule due to its role in intestinal homeostasis, barrier integrity, and inflammatory signaling. Emerging evidence suggests that altered Gal-4 ex-pression may reflect the progression of obesity-related inflammation, insulin resistance, and vascular pathology, highlighting its potential relevance in the early identification and stratification of cardiometabolic risk [52].

### 1.5. Galectin-4

Gal-4 was initially isolated from rat small intestine extracts and is now known as a 36 kDa protein containing two divergent, but structurally conserved carbohydrate-binding domains in one polypeptide chain [70]. The two carbohydrate recognition domains of Gal-4 comprise 133 and 130 amino acids, connected by a 34-amino-acid linker and preceded by a 17-amino-acid segment [71]. The CRD sequences share approximately 35% similarity, and the linker peptide has been shown to be particularly susceptible to proteolytic cleavage [70]. Due to the absence of a signal peptide for endoplasmic reticulum-mediated transport, Gal-4 is secreted via a non-classical pathway, which accounts for its presence not only within the cytoplasm, but also on the cell surface and in the extracellular milieu [72]. Gal-4 exhibits specific binding affinity for β-galactosides, including human blood group antigens, glycoproteins, mucin-like membrane proteins such as MUC1, glycosphingolipids and sulfated cholesterol [72]. The addition of a 3′-sulfate group to galactose and lactose enhances their binding affinity for Gal-4 [73].

Gal-4 contributes to the stabilization of lipid rafts by forming organized lattices with specific glycoproteins and glycolipids [74]. Gal-4 is capable of cross-linking a diverse array of glycolipids and brush border-associated proteins on the apical surface of enterocytes, such as aminpeptidase N and sucrose-isomaltase, which are commonly subject to proteolytic cleavage upon exposure to pancreatic proteases and lipases [64,75]. Through its simultaneous binding to membrane-associated glycolipids and brush boarder enzymes, Gal-4 prevents the proteolytic cleavage and subsequent release of the enzymes into the intestinal lumen [75]. Moreover, Gal-4 is essential for the recruitment of glycoproteins into lipid rafts and plays a crucial role in their apical trafficking by facilitating their sorting into carriers at the trans-Golgi network [76]. Gal-4 has been shown to promote epithelial cell migration toward sites of mucosal barrier disruption by interacting with the cadherin/catenin complex on the cell surface [77]. In addition, Gal-4 upregulates expression of cyclin B1, thereby promoting cell cycle progression and contributing to the replenishment, maturation and differentiation of epithelial cells, highlighting its role in maintaining intestinal epithelial integrity and supporting recovery of the impaired mucosal barrier [77].

The role of Gal-4 in gut microbiota homeostasis remains to be fully understood, since very little literature on this matter is available. Recent studies indicate that Gal-4 is a major factor contributing to host defense against pathogens in the gastrointestinal tract. Gal-4 binds to blood group B carbohydrates (O-antigen) expressed on the lipopolysaccharide chains of certain bacteria, including Escherichia coli, and induces bacterial death by disrupting membrane integrity [78]. Although Gal-4 recognizes blood group B antigens on human erythrocytes without compromising their membrane integrity, its antimicrobial activity extends to other surface carbohydrates such as α-1,3-galactose [78,79]. Intracellular Gal-4 in intestinal epithelial cells binds to cytosolic *Salmonella enterica* serovar Worthington and induces the formation of bacterial aggregates. Gal-4 constrains bacteria during their growth, reducing intracellular bacterial motility. Moreover, binding of Gal-4 to the O-antigen of *Salmonella enterica* serovar Worthington amplifies inflammasome formation by enhancing caspase-1 activation and production of IL-18 in infected intestinal epithelial cells [80].

In the context of intestinal inflammation driven by mutations in T cell receptor genes, Gal-4 functions as an activator of mucosal CD4^+^ T cells and exacerbates the inflammatory response by promoting interleukin-6 (IL-6) production, which in turn enhances CD4^+^ T cell survival [81]. Conversely, in a wild-type colitis model, Gal-4 also exhibits high-affinity binding to the CD3 complex on activated T cells, resulting in cell cycle inhibition and antigen-induced apoptosis, thereby contributing to the attenuation of experimental colitis through the elimination of mucosal T cells [82].

Gal-4 has been detected in a variety of malignancies, with most studies reporting alterations predominantly at the mRNA level, and has been implicated in the development, progression, and enhanced metastatic potential of hepatocellular carcinoma, as well as gastric, pancreatic, breast, colorectal, and lung cancers [83,84,85,86,87,88]. However, Gal-4 appears to play context-dependent and sometimes contradictory roles across different types of cancer cells [72].

Gal-4 plays a pivotal role in the structural and functional organization of the central nervous system by promoting axonal growth through the enhancement of the number and size of L1 neural cell adhesion molecule (NCAM L1) clusters on the axonal membrane [89]. Furthermore, Gal-4 contributes to the formation of myelin sheaths by negatively regulating oligodendrocytes differentiation and initiating myelination via modulation of myelin-associated gene expression [90,91].

The well-established roles of Gal-4 in processes such as inflammation and tumor progression, maintenance of intestinal epithelial integrity, central nervous system organization, myelin sheath formation, and its emerging role in antimicrobial defense are in common with several mechanisms that are increasingly recognized as key contributors to the pathogenesis of obesity, diabetes, and cardiovascular diseases, particularly inflammation and gut microbiota alterations. Given the central role of chronic low-grade inflammation and the emerging but still incompletely understood role of intestinal dysbiosis in metabolic and cardiovascular disorders, it may prove relevant to investigate the role of Gal-4 in this context. However, studies explicitly investigating the involvement of Gal-4 in obesity, type 2 diabetes, and cardiovascular disease remain limited. Therefore, this review aims to comprehensively summarize the current literature on Gal-4 in relation to these conditions.

## 2. Methods

**Figure 3:**
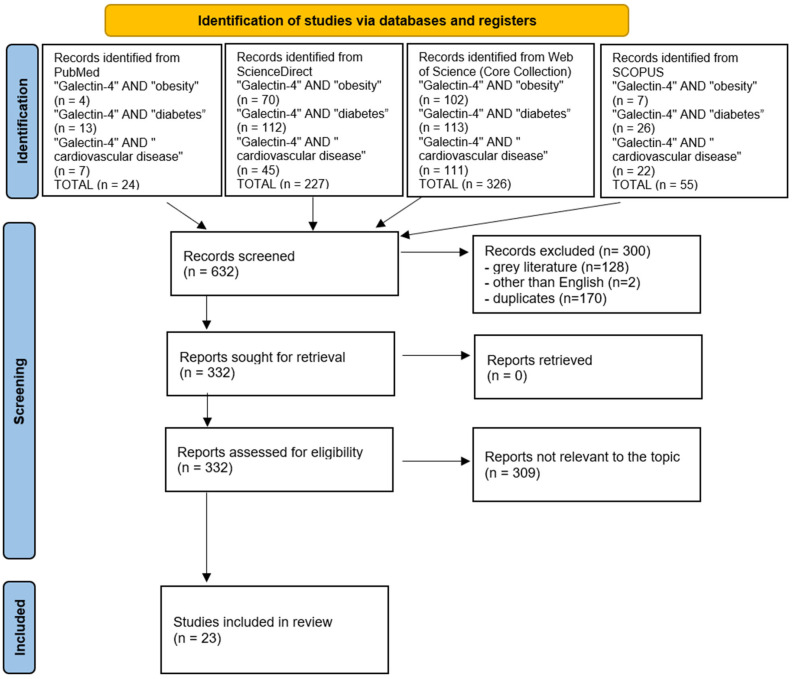
Schematic illustration of articles included in the review [92].

A comprehensive literature search was conducted between 15 and 30 June 2025 using four databases (PubMed, ScienceDirect, Web of Science (Core Collection) and SCOPUS) which capture the majority of peer-reviewed biomedical literature relevant to Galectin-4. The search employed the keywords: “Galectin-4” AND “obesity”, “Galectin-4” AND “diabetes”, and “Galectin-4” AND “cardiovascular disease”. To ensure relevance and quality, texts from the gray literature were excluded, and only full-text articles in English were considered. All retrieved articles were independently screened by both authors, with disagreements resolved by consensus. Studies that did not meet the inclusion criteria (manuscripts not containing data concerning Gal-4 or diseases described in this review) or presented biased outcomes were excluded. After this process, 23 studies met all criteria and were included for analysis (Figure 3, PRISMA diagram), providing a systematic and focused overview of the current evidence on the role of Gal-4 in obesity, diabetes, and cardiovascular diseases. Detailed study characteristics can be found in Supplementary Table S1.

This study was a Narrative Literature Review. Although it was not preregistered on platforms such as PROSPERO, we still referred to the PRISMA guidelines for reporting standards to enhance the transparency of the review.

## 3. Galectin-4 in Obesity, Diabetes, and Cardiovascular Diseases

The number of studies investigating the association between Gal-4 and obesity remains limited. The only existing reports on this relationship originate from the Malmö Preventive Project Re-Examination cohort (MPP-RES) and the HeArt and bRain failure inVESTigation trial (HARVEST- Malmö). Findings from the HARVEST-Malmö study demonstrated that elevated Gal-4 levels were significantly associated with a higher likelihood of obesity in heart failure patients; however, this association was observed exclusively among individuals with diabetes [93]. The MPP-RES study showed that in a cohort of middle-aged and older obese individuals, higher Gal-4 levels were independently associated with an increased likelihood of prior hospitalization. Notably, in stratified analysis, this association was statistically significant only among individuals with diabetes [94].

Numerous studies demonstrate that Gal-4 levels are significantly elevated in patients with diabetes compared to healthy individuals [93,94,95,96,97]. No significant differences in Gal-4 concentrations have been observed across diabetic heart failure patient subgroups stratified by age, sex, BMI, hypertension status, estimated glomerular filtration rate, or type of antidiabetic therapy, including both oral medication and insulin [96]. Interestingly, no interactions were observed between Gal-4 and diabetes in the endpoint analyses for incident coronary events and incident heart failure [98]. In vitro study of Gal-4–depleted HT-29 5M12 cells (enterocyte-like phenotype) showed a fourfold inhibition of dipeptidyl peptidase-4 (DPP-4) delivery to the apical membrane. DPP-4 is an enzyme responsible for the inactivation of glucose-dependent insulinotropic polypeptide (GIP) and glucagon-like peptide-1 (GLP-1) [99,100]. A hypothesis has been proposed that elevated Gal-4 expression enhances DPP-4 activity, thereby reducing GLP-1 activity and potentially contributing to an elevated risk of developing diabetes [95]. Furthermore, Gal-4 expression was examined in syncytiotrophoblasts (fetal placental tissue) and decidual cells (maternal placental tissue), revealing significantly elevated levels in the nuclei of syncytiotrophoblasts and in both nuclei and cytoplasm of decidual cells in placentas from women with gestational diabetes mellitus [101].

**Figure 4:**
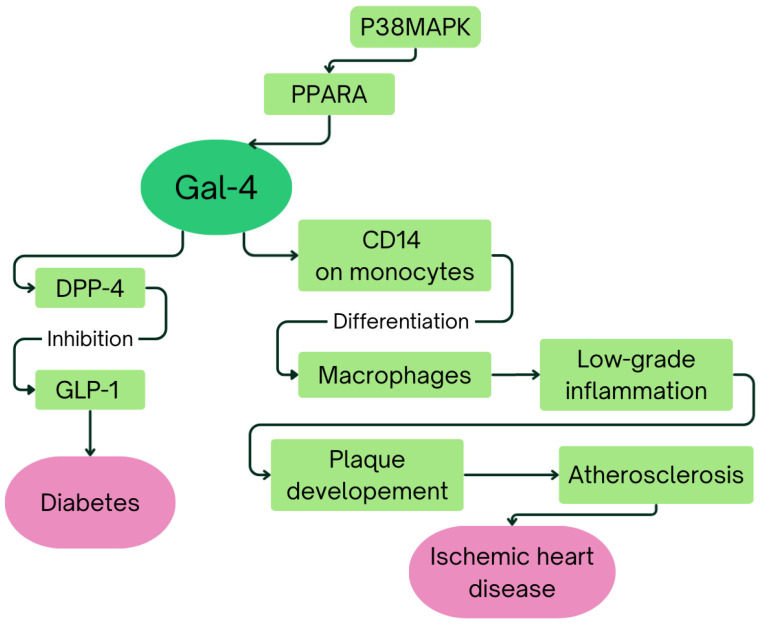
Schematic illustration of hypothetical mechanisms linking Gal-4 to diabetes and heart failure [95,99,100,102].

Recent studies have indicated that elevated Gal-4 concentrations are associated not only with prevalent and incident diabetes but also with an increased risk of future myocardial infarction, heart failure, and both cardiovascular and all-cause mortality [98]. In the German KORA F4 cohort, among five proteins initially linked to coronary heart disease (CHD), only Gal-4 maintained a significant association after full adjustment, whereas no significant relationship was observed with carotid intima-media thickness. The pathway analysis of CHD-associated proteins in KORA F4 study led to a hypothesis that activation of P38 mitogen-activated protein kinase (P38MAPK) leads to activation and phosphorylation of peroxisome proliferator activated receptor alpha (PPARA), which is predicted to directly increase expression of Gal-4 [102]. Moreover, Gal-4 binding to CD14 receptors on monocytes, thereby promoting their differentiation into macrophages, might contribute to the chronic low-grade inflammation observed in ischemic heart disease (IHD), subsequently facilitating plaque development and atherosclerosis. The fully adjusted HARVEST-Malmö study demonstrates a significant association between elevated Gal-4 concentrations and IHD. Furthermore, Galectins are suggested to play significant roles in cardiac fibrosis by stimulating myofibroblasts to produce extracellular matrix proteins, a key pathological mechanism involved in replacing damaged myocardial tissue during IHD progression [93]. Interestingly, Gal-4 upregulation has also been associated with diabetic aortic stenosis patients [103]. Moreover, significantly higher Gal-4 expression in heart failure patients correlates with diabetes, obesity, and ischemic heart failure etiology [93]. What is more, Gal-4 negatively correlates with coronary flow velocity reserve in patients with coronary microvascular dysfunction, which suggests its potential importance as a marker of coronary microcirculation and cardiac vascular function [104]. Figure 4 summarizes the hypothetical mechanisms linking Gal-4 to diabetes and heart failure.

The population-based LIFE Adult study identified seven proteins, Gal-4, MMP-4, GDF-15, JAM-A, PCSK9, vWF, and IL-1RT2, as positively associated with the presence of carotid plaques. Elevated plasma concentrations of Gal-4, PCSK9, and vWF were specifically linked to plaque occurrence in the common carotid artery, carotid bulb, and internal carotid artery. Moreover, the concentrations of all seven proteins correlated significantly with the total number of carotid plaques [105].

The Malmö Preventive Project, STANISLAS cohort, and HOMAGE case–control cohort demonstrated that elevated plasma concentrations of Gal-4, alongside ST2, GDF-15, and NT-proBNP, were associated with incident heart failure independent of established risk factors. However, these biomarkers were not linked to prevalent left ventricular hypertrophy or diastolic dysfunction. While NT-proBNP remains the strongest individual predictor, the inclusion of ST2, GDF-15, and Gal-4 improves the overall predictive accuracy for future heart failure [106]. Notably, in individuals with heart failure with reduced ejection fraction Gal-4 was significantly associated with diabetes, but was not in those with heart failure with preserved ejection fraction [93]. Significant correlation between Gal-4 concentrations and myocardial function, physical capacity, and daily physical activity were observed in comparative analysis of heart-failure patients and controls [107].

According to findings from the HOMAGE trial, plasma concentrations of Gal-4 were significantly elevated in patients with coronary artery disease. However, no statistically significant difference was observed in patients with a history of myocardial infarction [108]. Elevated Gal-4 levels were associated with the subsequent development of aortic valve stenosis requiring valve replacement, but this association was observed only among patients with coexisting coronary artery disease [109]. In a prospective repeated-measures study of patients with stable chronic heart failure, Gal-4 emerged as one of several biomarkers that exhibited a marked and progressive increase preceding adverse clinical events. Importantly, baseline levels of Gal-4, ST2, GDF-15, perlecan, and cystatin B were already significantly elevated in patients who later reached the primary endpoint compared to those who remained clinically stable [110]. Among phenotypically matched patients with ST-elevation myocardial infarction (STEMI) undergoing primary percutaneous coronary intervention, a relative rise in Gal-4 levels within the first 24 h post-procedure was independently associated with an increased risk of adverse outcomes, including all-cause mortality, heart failure or cardiogenic shock within 90 days [111].

Moreover, experimental studies in murine models demonstrate that Gal-4 is linked to the occurrence of ischemic stroke irrespective of presence of metabolic syndrome induced by a high-fat diet, aligning with observations form human cohort studies [112]. Furthermore, increased Gal-4 concentrations have been detected in association with symptomatic intracranial hemorrhagic transformation, a severe complication following ischemic stroke [113]. Additionally, studies in women with grade but no significant obstructive coronary artery disease have demonstrated strong associations between elevated Gal-4 concentrations and reduced myocardial blood flow reserve, suggesting the presence of coronary microvascular dysfunction that is independent of endothelial regulation [114]. Epidemiological studies have established that individuals living with HIV are at elevated risk for atherosclerotic cardiovascular disease. Proteomic analyses have shown that statin therapy in this population is associated with increased Gal-4 levels [115]. The key points of this section are summarized in Table 3.

## 4. Discussion

Current evidence on the association of Gal-4 with obesity comes solely from the population of a single Swedish city, Malmö, in two cohorts comprising 324 and 517 patients. Findings from these studies, namely association of Gal-4 concentrations with a higher likelihood of obesity (in patients with heart failure) and prior hospitalization being relevant only among individuals with diabetes, indicate that Gal-4 is not specific to obesity, but rather diabetes-related [93,94]. Furthermore, elevated Gal-4 reported concentrations in patients with obesity, diabetes and heart failure may only reflect the intensity of inflammation and fibrosis. Given the limited and homogeneous populations investigated, to draw any conclusions about the role of Gal-4 in obesity, further investigation is necessary.

Half of the studies providing evidence on the association of Gal-4 with diabetes focus on populations of heart failure patients, introducing additional complexity to the interpretation of these relationships [93,94,95,96,97,98,101,102,103,104,105,106,107,108,109,110,111,112,113,114,115]. The findings of this review should be interpreted with caution due to several methodological limitations of the available studies. Most of the evidence comes from observational designs, which are inherently susceptible to residual confounding, selection bias, and difficulties in establishing causal relationships. The available and cited publications are investigating associations between proteins and cardiometabolic diseases, thereby no conclusions can be drawn regarding causality. Moreover, there is no or little data available regarding factors such as socioeconomic status, duration of diabetes, the extent of atherosclerosis in stable and unstable CHD, and severity of other cardiovascular diseases, which may have influenced results. Furthermore, the included studies varied in terms of population characteristics, sample size, and outcome definitions, limiting the direct comparability of results. Almost all studies have been conducted in Northern Europe (Sweden, Norway, Denmark), as well as Germany and France, predominantly comprising individuals of European descent, which limits the generalizability of the findings. Population-specific factors such as genetic factors, dietary habits and microbiome profiles differ across global populations and must be taken into consideration. Due to the mostly cross-sectional nature of these studies and many comorbidities following obesity, diabetes and cardiovascular diseases, more focused analyses are needed to determine the relevance of Gal-4 as a biomarker of these conditions and provide insight into the complex molecular pathways involved. These limitations highlight the need for cautious interpretation of the current evidence and underscore the importance of future prospective and interventional studies.

## 5. Clinical Utility

Currently, Gal-4 does not possess any recognized clinical utility in the context of cardiometabolic diseases. Elhadad et al. reported a potential role of Gal-4 as a biomarker of CHD using ROC-AUC analyses in both KORA F3 and F4 cohorts [102]. However, no comparative evaluation regarding the sensitivity and specificity of Gal-4 in cardiometabolic conditions is currently available, the fact that inclusion of Gal-4, ST2, and GDF-15 as an additional parameters to NT-proBNP evaluation improves overall predictive accuracy for future heart failure may prove clinically significant in a multi-biomarker approach [106]. A relative rise in Gal-4 levels within the first 24 h among STEMI patients undergoing primary percutaneous coronary intervention was independently associated with an increased risk of adverse outcomes within 90 days and may contribute to improved post-procedural monitoring especially in a multi-biomarker approach. If the hypothesis that Gal-4 is linked to DPP-4 and GLP-1 activity, potentially contributing to the development of diabetes is correct, then inhibition of Gal-4 might reduce the risk of diabetes. However, this association remains speculative, given that Gal-4 is involved in trafficking of other vital molecules, lipid raft stabilization, CNS function, and other processes.

## 6. Conclusions

Gal-4 has recently emerged as a biomarker of interest in obesity, diabetes, and cardiovascular diseases, and the available literature remains very limited. Current evidence indicates consistently elevated circulating Gal-4 concentrations across these conditions; however, underlying molecular pathways and relationships have yet to be elucidated. At present, Gal-4 appears to hold prognostic value for disease severity and adverse clinical outcomes, but its potential as a biomarker or therapeutic target requires further investigation. Notably, the relationship between Gal-4 and obesity seems to be largely dependent on the presence of diabetes, indicating a complex interplay that warrants further investigation. That is why future studies should aim to clarify whether Gal-4 actively contributes to the pathogenesis of metabolic and cardiovascular diseases or reflects secondary processes such as inflammation, fibrosis, or immune dysregulation. A better understanding of Gal-4 biological function may not only define its role as a biomarker but also open novel strategies for targeted therapy in cardiometabolic disorders.

## Figures and Tables

**Table 1:** Diagnostic criteria for diabetes and prediabetes [35].

Condition	Fasting Plasma Glucose (FPG)	Oral Glucose Tolerance Test (OGTT)	Glycated Hemoglobin (HbA1C)	Random Plasma Glucose
Diabetes	≥126 mg/dL	2 h Plasma Glucose ≥ 200 mg/dL	≥6.5%	Classic symptoms of hyperglycemia and plasma glucose ≥ 200 mg/dL
Prediabetes	100–125 mg/dL	2 h Plasma Glucose 140–199 mg/dL	5.7–6.4%	-

**Table 2:** Classification of Galectins [52,61,64,65,66,67,68,69].

Galectin	Gene Symbol	Carbohydrate Preferential Affinity (β-d-Galactosides)	Organs Protein Expression
Galectin 1	LGALS1	LacNAc, poly-LacNAc, sulfated glycans	Bone marrow, brain, cervix, endometrium, lymph node, ovary, parathyroid gland, placenta, smooth muscle, skin, spleen, testis, tonsil, and vagina
Galectin 2	LGALS2	LacNAc, poly-LacNAc, lactose	Appendix, colon, duodenum, gallbladder, kidney, liver, lymph node, pancreas, rectum, small intestine, spleen, and tonsil
Galectin 3	LGALS3	LacNAc, poly-LacNAc, sulfated glycans, sialylated glycans	Adipose and soft tissue, bone marrow and lymphoid tissues, brain, endocrine tissues, female tissues, gastrointestinal tract, kidney and urinary bladder, lung, male tissues, muscle tissues, pancreas, proximal digestive tract, and skin
Galectin 3 binding protein	LGALS3BP	LacNAc, poly-LacNAc, lactose	Adipose and soft tissue, bone marrow and lymphoid tissues, brain, female tissues, gastrointestinal tract, kidney and urinary bladder, lung, male tissues, muscle tissues, proximal digestive tract, and skin
Galectin 4	LGALS4	LacNAc, lactose, sulfated glycans	Appendix, colon, duodenum, gallbladder, pancreas, rectum, small intestine, stomach, and neuronal cells
Galectin 7	LGALS7	LacNAc, sulfated glycans	Cervix (uterine), esophagus, oral mucosa, salivary gland, skin, tonsil, and vagina
Galectin 8	LGALS8	LacNAc, poly-LacNAc, 3′-*O*-sialylated and 3′-*O*-sulfated glycans	Adipose and soft tissue, bone marrow and lymphoid tissues, brain, endocrine tissues, female tissues, gastrointestinal tract, kidney and urinary bladder, lung, male tissues, muscle tissues, pancreas, proximal digestive tract, and skin
Galectin 9	LGALS9	LacNAc, poly-LacNAc, lactose, *N*-acetyl-LacNAc, fucosylated glycans	Adipose and soft tissue, bone marrow and lymphoid tissues, brain, endocrine tissues, female tissues, gastrointestinal tract, kidney and urinary bladder, lung, male tissues, muscle tissues, pancreas, proximal digestive tract, and skin
Galectin 9B	LGALS9B	LacNAc, poly-LacNAc	Appendix, bone marrow, breast, lymph node, spleen, and tonsil
Galectin 9C	LGALS9C	LacNAc, poly-LacNAc	Appendix, bronchus, colon, duodenum, gallbladder, lung, pancreas, spleen, stomach, and tonsil
Galectin 10	LGALS10	*N*-acetyl-d-glucosamine, d-mannose, weak to lactose	Lymph node, spleen, and tonsil
Galectin 12	LGALS12	β-d-galactose, lactose	Adipose tissue, low levels in heart, pancreas, spleen, and thymus
Galectin 13	LGALS13	LacNAc, *N*-acetyl-LacNAc, mannose, *N*-acetyl-galactosamine	Kidney, placenta, spleen, and urinary bladder
Placental Protein 13 (Galectin 14)	LGALS14	*N*-acetyl-LacNAc, sulfated glycans	Adrenal gland, colon, and kidney
Galectin 16	LGALS16	*N*-acetyl-LacNAc, β-d-galactose, lactose	Placenta

**Table 3:** The key points of Galectin-4 in obesity, diabetes, and cardiovascular diseases.

GAL-4 Summary	Obesity	Diabetes	Cardiovascular Diseases
Available Studies	1. HeArt and bRain failure inVESTigation trial (HARVEST- Malmö) [93]. 2. Malmö Preventive Project Re-Examination Study (MPP-RES) [94].	1. HeArt and bRain failure inVESTigation trial (HARVEST- Malmö) [93]. 2. Malmö Preventive Project Re-Examination Study (MPP-RES) [94,95,98]. 3. A Systems Biology Study to Tailored Treatment in Chronic Heart Failure (BIOSTAT-CHF) [96]. 4. EpiHealth cohort [97]. 5. Other studies [101].	1. HeArt and bRain failure inVESTigation trial (HARVEST- Malmö) [93]. 2. Malmö Preventive Project Re-Examination Study (MPP-RES) [94,98,106] 3. A Systems Biology Study to Tailored Treatment in Chronic Heart Failure (BIOSTAT-CHF) [96]. 4. KORA F4 cohort [102]. 5. LIFE-Adult study [105]. 6. STANISLAS cohort [106]. 7. HOMAGE case–control cohort [106,108]. 8. Other studies [103,104,107,109,110,111,112,113,114,115].
GAL-4 Concentrations	1. Among middle-aged and older obese individuals elevated Galectin-4 concentrations correlate with an increased probability of prior hospitalization [94]. 2. Elevated Galectin-4 concentrations correlate with higher likelihood of obesity among heart failure patients [93].	1. Galectin-4 levels are significantly elevated in patients with prevalent and incident diabetes compared to healthy individuals [93,94,95,96,97]. 2. Galectin-4 levels are significantly elevated in the nuclei of syncytiotrophoblasts and in both nuclei and cytoplasm of decidual cells in placentas from women with gestational diabetes mellitus [101].	Elevated Galectin-4 concentrations are observed in patients with: 1. Coronary heart disease [93,102,108]. 2. Diabetic aortic stenosis [103]. 3. Heart failure [93]. 4. Coronary microvascular dysfunction [104]. 5. Carotid plaques [105]. 6. Ischemic stroke [112]. 7. Symptomatic intracranial hemorrhagic transformation [113].
Additional Information	Both associations were statistically significant only among individuals with diabetes, meaning that the relationship between elevated Galectin-4 levels and an increased risk of obesity or prior hospitalization is primarily driven by the presence of diabetes [93,94].	No significant differences in Galectin-4 concentrations have been observed across diabetic patient subgroups stratified by age, sex, BMI, hypertension status, estimated glomerular filtration rate, or type of antidiabetic therapy, including both oral medication and insulin [96].	In patients with stable chronic heart failure, Galectin-4 emerged as one of several biomarkers that exhibited a marked and progressive increase preceding adverse clinical events. Baseline levels of Galectin-4, ST2, GDF-15, perlecan, and cystatin B were already significantly elevated in patients who later reached the primary endpoint [110].

## Data Availability

No new data were created or analyzed in this study.

## Supplementary Materials

The following supporting information can be downloaded at: https://www.mdpi.com/article/10.3390/ijms26199402/s1.
