# Supplementary material for: Association of Elevated Galectin-4 Concentrations with Obesity, Diabetes, and Cardiovascular Diseases
Source: Int J Mol Sci. 2025 Sep 26;26(19):9402. doi: 10.3390/ijms26199402 (PMC12524632; doi:10.3390/ijms26199402)
Supplement: Supplementary file 1 [file ijms-26-09402-s001.zip › ijms-3880315-supplementary.pdf]

Supplementary Table S1. Comprehensive summary of study characteristics for discussed manuscripts.

Study (First Author, Year)	Design	Population	Sample Size	Assay/Platform	Matrix	Gal-4 Distribution	Endpoints	Covariates	Effect Size (OR/HR, 95% CI)
Dieden, 2023	Cross-sectional	HF patients, Sweden	323	Olink CVD III	Plasma	Higher in DM/obesity	Prevalent DM, obesity	Age, sex, BMI, eGFR, NT-proBNP	DM: OR 2.60 (1.56–4.32); Obesity in DM: OR 2.48 (1.09–5.62)
Korduner, 2022	Cross-sectional	Obese adults	517	Olink CVD III	Plasma	Higher in hospitalized obese	Hospitalization	Age, sex, risk factors	OR 1.72 (1.16–2.54); DM: OR 2.26 (1.25–4.07)
Molvin, 2019	Prospective cohort	Malmö Preventive Project	1026	Olink CVD III	Plasma	Higher in incident DM	Incident DM	Age, sex, BMI, glucose	HR 1.373 (1.147–1.643); p=0.00054
Tromp, 2020	Cross-sectional	HF ± DM	1572	Olink CVD III	Plasma	Higher in DM	Biomarker profiles, outcomes	Age, sex, risk model	p<0.05; FDR <0.05
Beijer, 2019	Cross-sectional	Diabetes, proteomics/genotyping	2467	Olink CVD III	Plasma	Higher in DM	Causal pathways in DM	Age, sex, BMI, glucose	OR 1.69 (1.28, 2.25)
Molvin, 2020	Cross-sectional	DM, CVD	1713	Olink CVD III	Plasma	Higher in ACM, CVM, ICEs, IHF	ACM, CVM, ICEs, IHF	Demographics, Clinical profile, Medical history	ACM: HR 1.29 (1.17 1.41)<br>CVM: HR 1.38 (1.22 1.56)<br>ICEs: HR 1.34 (1.14 1.57)<br>IHF: HR 1.26 (1.03 1.54)
Delacour, 2005	Experimental	Enterocyte-like cells	NR	Confocal microscopy, Immunoprecipitation, Western blot, retroviral-mediated RNAi	HT-29 5M12 Cells	NR	Membrane trafficking	NR	NR
Schrader, 2022	Cross-sectional	Gestational DM placentas	80	Immunofluorescence double staining	Cells	Overexpression in GDM	GDM	NR	NR
Elhadad, 2024	Case-control	KORA F4/F3	1000	SOMAscan/ELISA	Plasma	Higher in CHD	CHD, CIMT	Age, sex, BMI, DM, lipids	CHD: OR 1.58 (1.30–1.93); Replication: OR 1.40 (1.09–1.88)
Lee, 2023	Prospective cohort	Severe AS, Korea	253/100	Olink CVD III	Plasma	Higher in DM	HF admission, death	Clinical covariates, AVR	HR 1.88 (1.06–3.31)
Schroder, 2019	Cross-sectional	Women, angina, no CAD, CMD	174	Olink CVD III	NR	Higher in CMD	CFVR	Age	p=0.026 (correlation)
Garcia, 2024	Population-based	LIFE-Adult study	2024	Olink CVD III	Plasma	Higher in plaque presence	Subclinical CVD traits	27 covariates including eGFR, triglycerides, sex	p=0.0017 (correlation)
Dieden, 2024	Cohort	3 cohorts, HF	>1500	Olink CVD III	Plasma	Higher in HF	LVH, DD, incident HF	Age, sex, risk factors	Gal-4 associated with incident HF
Rullman, 2020	Cross-sectional	Severe HF	66	Olink CVD III	Plasma	Higher in HF	Function, prognosis	Age, eGFR, peakVO2 and LVEF	p=0.002 (correlation)

Supplementary Table S1. Comprehensive summary of study characteristics for discussed manuscripts.

Study (First Author, Year)	Design	Population	Sample Size	Assay/Platform	Matrix	Gal-4 Distribution	Endpoints	Covariates	Effect Size (OR/HR, 95% CI)
Santos-Ferreira, 2024	Cohort	IHD at risk for HF	527	Olink CVD III	Plasma	Higher in CAD	HF risk	Sex, age, diabetes, eGFR, risk factors	CAD: OR 3.06 (1.66, 5.83)
Ljungberg, 2018	Cohort	Incident AS	334	Olink CVD III	Plasma	Higher in future AVR with CAD	AS requiring replacement	Age, sex, BMI, CAD	OR 1.27 (1.05, 1.55)
Bouwens, 2019	Cohort	Chronic HF	263	Olink CVD III	Plasma	Higher levels at baseline in patients who later experienced the end point	Cardiac remodeling	Age, sex, clinical characteristics, HF medication, NT-proBNP	HR 1.18 (1.05 1.36)
Shavadia, 2022	Case-control	STEMI	139	Olink CVD III	Serum	Relative difference in concentrations and death, shock and heart failure within 90 days	Outcomes	Age, sex, BMI, clinical history	HR 1.16 (1.05–1.30)
Jujic, 2023	Cross-sectional/animal	Human, mice	1737	Olink CVD III	Plasma	Higher in stroke	Prevalent stroke	Age, sex, cardiometabolic	OR 1.52 (1.01–2.30)
Yuan, 2025	Retrospective/ prospective	Ischemic stroke	304	Olink PEA	Serum	Higher in s-HT vs non-s-HT	Hemorrhagic transformation	Age, sex, medical history, treatment	p=0.0205 (correlation)
Schroder, 2018	Cross-sectional	Women, angina, no CAD, CMD	97	Olink CVD III	Plasma	Higher in CMD	MBFR	Age	p=0.008 (correlation)
Toribio, 2018	RCT	HIV, statin trial	225	Olink CVD III	Plasma	Higher in HIV statin therapy	CVD pathways	Age, sex, ethnicity, BMI, eGFR	p=0.0006 (correlation)

Abbreviations: ACM - All-cause mortality; AS - Aortic stenosis; AVR - Aortic valve replacement; BMI - Body mass index; CAD - Coronary artery disease; CFVR - Coronary flow velocity reserve; CMR - Cardiac magnetic resonance (imaging); CMD - Coronary microvascular dysfunction; CHD - Coronary heart disease; CIMT - Carotid intima-media thickness; CVD - Cardiovascular disease; CVM - Cardiovascular mortality; DD - Diastolic dysfunction; DM - Diabetes mellitus; ECV - Extracellular volume fraction (from CMR, marker of diffuse myocardial fibrosis); eGFR - Estimated glomerular filtration rate; FPG - Fasting plasma glucose; GDF15 - Growth differentiation factor 15; GIP - Glucose-dependent insulinotropic polypeptide; HDL - High-density lipoprotein cholesterol; HF - Heart failure; HO - Hospitalized subjects with obesity; HR - Hazard ratio; ICEs - Incident coronary events; IGFBP-1/2/7 - Insulin-like growth factor-binding protein 1/2/7; IHD - Ischemic heart disease; LDL - Low-density lipoprotein cholesterol; LGE - Late gadolinium enhancement (CMR marker of replacement fibrosis); LVEF - Left ventricular ejection fraction; LVH - Left ventricular hypertrophy; MBFR - Myocardial blood flow reserve; NHO - Non-hospitalized subjects with obesity; NT-proBNP - N-terminal pro-B-type natriuretic peptide (heart failure biomarker); NR – not reported; OR - Odds ratio; PEA - Proximity extension assay (Olink proteomics platform); PET - Positron emission tomography; SBP - Systolic blood pressure; s-HT - Symptomatic intracranial hemorrhage transformation; STEMI - ST-segment elevation myocardial infarction; tPA - Tissue-type plasminogen activator; vWF - von Willebrand factor
